# A proposed method for estimating habitat suitability of weed biological control agents with experimentally derived thermal injury and weather data

**DOI:** 10.1093/ee/nvaf099

**Published:** 2025-10-01

**Authors:** Ian A Knight, Felix E Bingham, Megann M Harlow, Annie H Huang, Chelsea Bohaty, Nathan E Harms

**Affiliations:** Aquatic Ecology and Invasive Species Branch, US Army Engineer Research and Development Center, Vicksburg, MS, USA; Aquatic Ecology and Invasive Species Branch, US Army Engineer Research and Development Center, Vicksburg, MS, USA; Aquatic Ecology and Invasive Species Branch, US Army Engineer Research and Development Center, Lewisville, TX, USA; Oak Ridge Institute for Science and Education, Department of Energy, Vicksburg, MS, USA; Jacksonville District, US Army Corps of Engineers Jacksonville District, FL, USA; Aquatic Ecology and Invasive Species Branch, US Army Engineer Research and Development Center, Lewisville, TX, USA

**Keywords:** species distribution modeling, cold-tolerance, climatic mismatch, biological control

## Abstract

Ecological niche modelling provides a tool for making *a priori* predictions of habitat suitability for biological control agents. Current approaches may be limited by available data but improved by the incorporation of physiological data. Alligatorweed, *Alternanthera philoxeroides* (Mart.) Griseb. (Caryophyllales: Ameranthaceae), is controlled across much of its introduced range in the United States of America by the alligatorweed flea beetle, *Agasicles hygrophila* Selman and Vogt (Coleoptera: Chrysomelidae); however, insufficient control is observed at temperate latitudes. Investigations into alligatorweed thrips, *Amynothrips andersoni* O’Neill (Thysanoptera: Phlaeothripidae), indicate that they are more cold-tolerant with a broader predicted range. The upper limit of the chill injury zone (ULCIZ) and the sum of injurious temperatures (SIT) are measures that can be used to compare relative cold tolerance among biocontrol agents. Here we propose a method for integrating these parameters with weather data to predict overwintering mortality. The ULCIZ and SIT of *Am. andersoni* and *Ag. hygrophila* were experimentally determined, then habitat suitability for each species was modeled using the proposed method and 20 yr of weather data. ULCIZ was −2.94 and 4.52 °C, and SIT was 307.19 and 251.27 for *Am. andersoni* and *Ag. hygrophila*, respectively, indicating that *Am. andersoni* begins accumulating chill injury at a lower temperature than *Ag. hygrophila* and does so at a slower rate. Using this method, 91.8% of *Al. philoxeroides’s* range in the USA was predicted to fall within highly or moderately suitable habitat for *Am. andersoni*, compared to 15.9% for *Ag. hygrophila*. Ranges predicted by the proposed method are similar to previous correlative ENMs.

## Introduction

Climatic mismatches between invasive species and their biological control agents result in reduced efficacy or complete failure of biological control programs. The issue has been receiving increased attention in recent years, particularly with respect to otherwise historically successful biological programs of invasive aquatic weeds (reviewed in Harms et al. 2021). A traditional solution to the problem is a return to foreign exploration for new agent genotypes or species better adapted to the mismatched regions (Russell et al. 2017). A less traditional approach for widely distributed agents, conducting local population surveys across an agent’s introduced range may yield the identification of intraspecific differences in traits that make them more suitable (Reddy et al. 2019, Knight et al. 2023). In either instance, particularly for new biological control programs, a method for predicting habitat suitability would be a valuable tool to promote the success of selected agents.

Multiple species distribution modelling approaches exist to predict species distributions based on climatic data. Ecological Niche Modelling (ENM) uses the known distribution of presence-absence or abundance of a species with environmental covariates to estimate habitat suitability (Elith and Leathwick 2009). In the context of biological control, this approach provides a relatively low-cost tool for predicting the potential ranges of invasive species and their natural enemies in their introduced ranges and for informing foreign exploration for new biological control agents (Mukherjee et al. 2014, Russell et al. 2017). However, predictions can be of limited utility when either occurrence data are low (Pearson et al. 2007) or species do not fill their fundamental niche within their native range (see also Tingley et al. 2014, Evans et al. 2015). In contrast, mechanistic modelling uses empirically determined environmental tolerances to predict species’ distributions (reviewed in Kearney and Porter 2009). Similarly, this approach has been used to model the predicted distributions of biological control agents within their introduced ranges, generally relying on temperature-dependent developmental rates to determine lower developmental thresholds and estimate a number of generations per year (eg Manrique et al. 2008, May and Coetzee 2013). Further, approaches exist that attempt to blend ENMs and mechanistic models, known as hybrid or fitted process-based models (see Dormann et al. 2012).

Alligatorweed, *Alternanthera philoxeroides* (Mart.) Briseb. (Caryophyllales: Amaranthaceae), is a globally significant invasive plant species. In 1960’s and 70’s, alligatorweed was the target of a very successful biological control program in the southeastern United States of America, with the majority of the success attributed to the alligatorweed flea beetle, *Agasicles hygrophila* Selman and Vogt (Coleoptera: Chysomelidae) (Spencer and Coulson 1976). In subsequent decades, however, insufficient control was observed in temperate regions, and unsuccessful attempts were made to source additional agents from more temperate areas of the native range (Buckingham and Boucias 1982). Modelling by Julien (1995) indicated that *Al. philoxeroides* had a greater potential range than that of *Ag. hygrophila*. Additionally, alligatorweed thrips, *Amynothrips andersoni* O’Neill (Thysanoptera: Phlaeothripidae), were also introduced as part of the same program, though not as widely (Spencer and Coulson 1976). During subsequent efforts to improve biological control of *Al. philoxeroides* in temperate regions of the USA, the thrips were found to be significantly more cold-tolerant with increased potential for *Al. philoxeroides* control in temperate regions (Knight and Harms 2022). A recent ENM by Schmid et al. (2025) suggests that the predicted range of *Am. andersoni* is more similar to *Al. philoxeroides* than *Ag. hygrophila*, while simultaneously supporting the original *Ag. hygrophila* predictions by Julien (1995); however, the number of records for *Am. andersoni* was relatively low (*n* = 35), a common limitation of ENMs.

Though modelling the realized niche of biological control agents is useful for understanding implementation of biological control programs, the *Al. philoxeroides* system highlights the need for an *a priori* method of estimating habitat suitability when the fundamental niche of a potential agent is not well known. Mechanistic modelling provides a tool for making these estimations, though many commonly used parameters have their own limitations and considerations (reviewed in Evans et al. 2015). The use of lower thermal limits, as may be most relevant in the case of many natural enemies of tropical weeds in temperate climates (Harms et al. 2021), is limited by the relationship between temperature, exposure time, and chill injury (Overgaard et al. 2014). The time-temperature modelling approach developed by Nedve˘D et al. (1998) provided a potential solution by calculating the parameters that describe the threshold below which chill injury accumulates, the Upper Limit of Chill Injury Zone (ULCIZ), and the relationship between temperature and exposure time, the Sum of Injurious Temperatures (SIT).

There have been a few attempts to find an appropriate method for incorporating these parameters into range estimates. Earlier attempts simply used the ULCIZ to identify an isocline that delineates an intermediate zone of suitability (eg Zhao et al. 2015). Wang et al. (2019) took this approach a step farther, using climate data to identify severe chilling events and calculating estimated mortality based on the temperature and duration of the event. No approach to date has used ULCIZ and SIT to estimate chill mortality over time directly from temperature data. Here we describe the determination of the ULCIZ of *Ag. hygrophila* and *Am. andersoni* and propose a method of estimating cumulative chill injury and resulting mortality from species-specific ULCIZ. We use measured weather data in an approach that builds on the methods of Wang et al. (2019), then use data from weather stations from across the continental US to extrapolate estimated mortality of both species relative to the known distribution of *Al. philoxeroides*. We go one step further by using the model we developed for *A. hygrophila* to conduct a retrospective analysis of a long-term biological control distribution program in the southeastern USA. We compare our estimates about winter mortality to the availability of agents in the following spring.

## Materials and Methods

### Source Populations and Culturing

*Amynothrips andersoni* and *Agasicles hygrophila*, used for chill injury assays, were reared at the US Army Engineer Research Development Center (ERDC) (Vicksburg, Mississippi). The source of *Am. andersoni* populations used in this study and the culture methods used to maintain the colony are described in Harms et al. (2025). The populations described therein were combined into a single culture 6 mo prior to their use in chill tolerance experiments, which were conducted from December 2023 through January 2024. The source of the *Ag. hygrophila* populations used in this study and the culture methods are described in Knight et al. (2023). Populations were maintained and assayed separately between February and October 2021, though results are pooled. All individuals were collected between approximately 1 to 2 wk from emergence for use in experiments.

### Experimental Methods

*Amynothrips andersoni* chill tolerance experiments were incomplete cross 2-way factorial designs with temperature and exposure duration as the independent variables. A single experimental unit consisted of ten recently emerged adult *Am. andersoni*. Individuals were collected using a fine paintbrush and distributed to a petri dish (9 cm diameter). Each petri dish also contained a small clipping of moistened paper towel and a single leaf of *Al. philoxeroides*. The petri dish was sealed with parafilm once the thrips had been added. Temperature treatments were -12, −9, −6, −3, 0, and 3 °C, and exposure time treatments were 6, 12, 24, 48, 96, 192, and 384 h. Combinations of −12 and −9 °C at exposure times greater than 96 h and −6 and −3 °C at exposure times less than 24 h were omitted to reduce the number of replicates needed while still spanning the full range of effectual temperature and time treatments. Each treatment combination contained 5 replicates for a total of 50 adult thrips per treatment. A total of 1,600 adult thrips were used for the experiment.

*Agasicles hygrophila* chill tolerance experiments were a complete 2-way factorial design. A single experimental unit consisted of a single adult beetle, collected using soft forceps, and placed in a 946 ml plastic deli cup with 2 cm of moist sand into which a single 10 cm shoot of *Al. philoxeroides* was inserted. Cups were sealed with Dacron Chiffon netting (∼240 µ mesh size) and rubber bands. Temperature treatments were −6, −2, 0, and 2 °C, and exposure time treatments were 6, 12, 24, 48, and 96 h. Each treatment combination contained a minimum of 7 replicates per population assayed, though more replicates were used when sufficient beetles were available. A total of 1,358 adult beetles were used for this experiment.

To begin the experiment, units from each replicate were distributed to their respective treatment temperature chambers (Model LT-41VL, Percival Scientific, Perry, Iowa, United States) and the temperature ramping program was initiated. Each program was set to ramp down from 23 °C, to the target temperature at a rate of 0.25 °C/min. The chambers held their target temperature once reached, which initiated the start time of each exposure-duration treatment. Chamber photoperiod was set to 14:10 L:D. When a treatment exposure duration was reached, those replicates were removed from the associated chamber and transferred to a 23 °C chamber for 24 h before survival was assessed. Survival was determined by gently touching insects with the tip of a paintbrush to stimulate movement if alive. Individual *Ag. hygrophila* were recorded as either alive or dead, and the proportion of live *Am. andersoni* was recorded for each petri dish.

### Upper Limit of Chill Injury Zone

The combined effect of low temperature and exposure time on agent survival was modeled following the approach described in Nedve˘D et al. (1998):

$$S(t,\;T)=\frac{e^{a+bt(T-c)}}{1+e^{a+bt(T-c)}}$$

S(t, T) is the estimated survival of the population after a duration (t; hours) of exposure at a given temperature (T; °C). The parameter c is the upper limit of the chill injury zone (ULCIZ), the lowest temperature that does not result in chill injury. The ratio of a/b describes the time-temperature relationship with respect to chill injury. Parameters for chill injury models were estimated using nonlinear least squares regression with the nlme package in R (R version 4.3.2, R Core Team, Vienna, Austria).

### Estimation of Cumulative Mortality

To estimate cumulative mortality over time (M_cumulative_) as a result of chill injury in response to a fluctuating temperature we developed the following approach:

Mcumulative=∑{Mi,0,Ti≤UCLIZ Ti>UCLIZ

$$M_{i}=\;S_{i-1}-S_{i}$$

$$S_{i}=\;\frac{e^{a+bt_{i}(T_{i}-c)}}{{1+e}^{a+bt_{i}(T_{i}-c)}}$$

$$t_{i}=\;\frac{\ln\left(\frac{S_{i-1}}{1-S_{i-1}}\right)-a}{b(T_{i}-c)}+x$$

where M_i_ is the incremental mortality at time increment *i*, *S_i_* is the estimated survival at time increment *i*, *S_i_*_-1_ is the estimated survival at the time increment preceding *S_i_* and *T_i_* is the measured temperature at time increment *i*. When time increment *i* is equal to 1 (the first observation in the series), *S_i_*_-1_ is equal to the intercept of equation 1 where time equals 0:

$$S(0,\;T)=\frac{e^{a}}{1+e^{a}}$$

Because exposure to injurious environmental temperatures is not consistent in the field, time increment *i* (*t_i_*) is estimated for each increment rather than taken as the specific time in the series. The constant *x* is the time resolution of the temperature dataset relative to the time resolution of the survival data used to calculate the parameters a, b, and c. For example, if the time resolution of the survival dataset was measured in hours and the temperature dataset is recorded in hours, then *x* is 1; if the survival data set was measured in hours and the temperature dataset recorded in minutes, then *x* is 1/60.

### Temperature Datasets

Twenty years (2002 to 2022) of 1-min surface temperature data was obtained from the National Oceanic and Atmospheric Administration (NOAA) Automated Surface Observations System (ASOS) (ftp://ftp.ncdc.noaa.gov/pub/data/asos-onemin/). Raw data were cleaned with Python using the Jupyter Notebook (Kluyver et al. 2016) following Wang et al. (2019). Data were aggregated for each station location and organized into single-year files beginning in July and ending in June of the following year. Weather stations were excluded from the analysis if they were outside the contiguous United States, missing greater than 25% of data from months between October and April, or had fewer than 10 yr of data. The final dataset contained 749 weather stations and was used to estimate monthly cumulative mortality due to chill injury for each file using the approach described above. For each weather station, mortality from the month with the highest estimated mortality was selected for each year and used to estimate the mean and standard deviation in winter mortality.

Maps of predicted mortality were generated using mean estimated winter mortality for each species for the contiguous United States. Mortality values were interpolated using Ordinary Kriging with a spherical semi-variogram model and variable search radius of 12 points in ArcGIS Pro (ESRI, Redlands, California, United States). Predicted mortality values were binned into 5 suitability categories: areas where predicted mortality was <25% were considered highly suitable, 26% to 50% were moderately suitable, 51% to 75% were moderately unsuitable, 75% to 95% were highly unsuitable, and >96% were completely unsuitable. The range of *Al. philoxeroides* in the United States was mapped using historical occurrence data (EDDMapS 2025). *Alternanthera philoxeroides* occurrence records were then thinned in ArcGIS Pro to a radius of 15 km, resulting in 557 records spread across the southeastern United States and California (S1). Predicted mortality values for *Am. andersoni* and *Ag. hygrophila* were extracted to the *Al. philoxeroides* occurrence data to predict the relative suitability of each species for the extent of alligatorweed in the United States.

### *Winter Temperature and the* Ag. hygrophila *Annual Redistribution Program*

Since 1981, the Jacksonville District of the US Army Corps of Engineers has operates an Alligator weed [sic] Biocontrol Project, which collects and redistributes *Ag. hygrophila* annually to various agencies across the USA (Bohaty 2020). Collections are made in waterways of north-central Florida each spring, typically over a 2-wk period each year. Collection data, including annual collection location and estimated number of beetles collected were obtained. Interpolated temperature estimates were generated for the state of Florida as described above with the exception that instead of calculating mean estimated mortality over several years, mortality was estimated separately for each year of the collection program. Annual estimated mortality was paired with spring collection data to assess the relationship between variable winter temperatures and success of the beetle collection program. Years when no collections were made, the collection location was unknown, or that occurred before 2002 were omitted from this analysis.

### Statistical Analysis

The time-temperature relationship with mortality for *Am. andersoni* and pooled *Ag. hygrophila* data was modeled using logistic regression in SAS (SAS Institute, Cary, North Carolina, United States), with binomial and binary error distributions, respectively. Linear regression of *Ag. hygrophila* collection numbers and annual collection site mortality estimates was done in linear regression in SAS.

## Results

Both time and temperature affected mortality for *Am. andersoni* and *Ag. hygrophila*. For every hour increase in exposure time, the log-odds of mortality increase by 0.0048 ± 0.0018 (*P* = 0.010) for *Am. andersoni* and 0.044 ± 0.004 (*P* < 0.001) for *Ag. hygrophila*. Similarly, for every degree (°C) drop in temperature, the log-odds of mortality increased by 0.161 ± 0.052 (*P* = 0.002) and 0.203 ± 0.049 (*P* < 0.001) for *Am. andersoni* and *Ag. hygrophila*, respectively. Though not directly comparable, *Am. andersoni* had lower mortality at similar temperatures and exposure times relative to *Ag. hygrophila* (Tables 1 and 2).

**Table 1.** Mean (±SE) mortality of *Amynothrips andersoni* by temperature and exposure time

Time (h)	3°C	0°C	−3°C	−6°C	−9°C	−12°C
6	.	.	0.098 ± 0.133	0.24 ± 0.191	0.351 ± 0.213	0.251 ± 0.194
12	.	.	0.02 ± 0.063	0.038 ± 0.086	0.361 ± 0.215	0.58 ± 0.221
24	0.547 ± 0.223	0.036 ± 0.084	0 ± 0.001	0.113 ± 0.142	0.253 ± 0.194	0.96 ± 0.088
48	0.35 ± 0.213	0.084 ± 0.124	0 ± 0.001	0.191 ± 0.176	0.239 ± 0.191	1 ± 0.001
96	0.5 ± 0.224	0.06 ± 0.106	0 ± 0.001	0.24 ± 0.191	0.6 ± 0.346	1 ± 0.001
192	0.723 ± 0.2	0.06 ± 0.106	0.164 ± 0.166	0.689 ± 0.207	.	.
384	0.751 ± 0.194	0 ± 0.001	0.4 ± 0.219	.	.	.

**Table 2.** Mean (±SE) mortality of *Agasicles hygrophila* by temperature and exposure time

Time (h)	2°C	−2°C	−4°C	−6°C
6	0.016 ± 0.016	0.05 ± 0.028	0.014 ± 0.014	0.111 ± 0.037
12	0.016 ± 0.016	0.203 ± 0.05	0.125 ± 0.039	0.338 ± 0.056
24	0.016 ± 0.016	0.333 ± 0.059	0.253 ± 0.049	0.62 ± 0.058
48	0.232 ± 0.056	0.554 ± 0.066	0.845 ± 0.043	0.957 ± 0.024
96	0.367 ± 0.066	0.922 ± 0.034	0.859 ± 0.037	1 ± 0

**Fig. 1.**
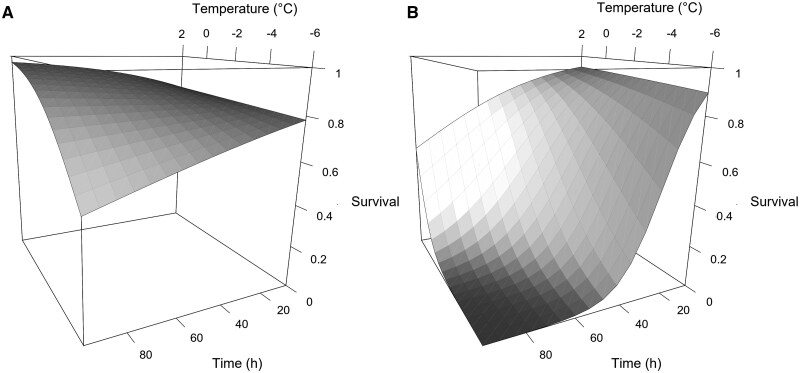
Surface plot of estimated survival (1 - mortality) as a function of temperature and exposure time for A) *Amynothrips andersoni* and B) *Agasicles hygrophila*.

The temperature at which *Am. andersoni* began accumulating chill injury (ie upper limit of the chill injury zone) was 7.46 °C lower than *Ag. hygrophila* (Table 3). The sum of injurious temperatures was 307.19 for *Am. andersoni* and 251.27 for *Ag. hygrophila*, indicating that *Ag. hygrophila* accumulates chill injury more rapidly than *Am. andersoni*. This relationship can be visualized using a 3-dimensional surface plot (Fig. 1) but may be simplified when survival is set to 0.5 using the equation described by Nedve˘D et al. (1998):

$$t=\;-\frac{a}{b}(\frac{1}{T-c})$$

**Table 3.** Upper limit of chill injury zone parameter estimates (±2*SD; 95% CI) for *Amynothrips andersoni* and *Agasicles hygrophila*, determined through nonlinear least squares regression

Parameter	*Am. andersoni*	*Ag. hygrophila*
a	1.367 ± 0.348	2.895 ± 0.325
b	4.45e-3 ± 1.36e-3	0.01152 ± 0.00169
c	–2.941 ± 0.717	4.520 ± 0.411

Using this equation, the difference in SIT between the 2 species indicates that the time required to reach 50% survival is 55.92 h shorter for *Ag. hygrophila* for each degree (°C) below their respective ULCIZ values. Not only does *Am. andersoni* begin accumulating chill injury at a lower temperature, but the rate of accumulation is slower.

**Fig. 2.**
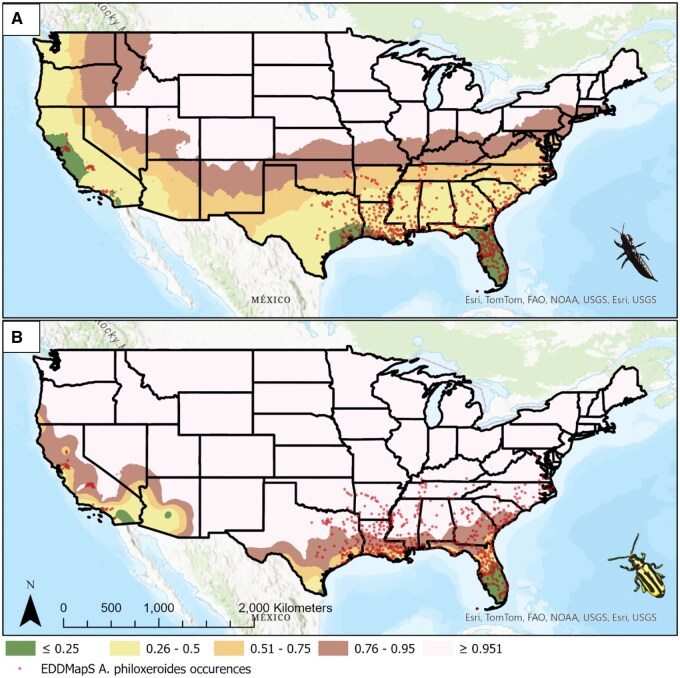
Estimated peak monthly winter mortality for A) *Amynothrips andersoni* and B) *Agasicles hygrophila*. Predicted mortality of 0.25 was considered highly suitable, 0.26 to 0.50 were moderately suitable, 0.51 to 0.75 were moderately unsuitable, 0.75 to 0.95 were highly unsuitable, and > 0.96 was completely unsuitable.

Calculation of peak winter mortality using the ASOS climate data revealed stark differences in predicted suitable habitat for each species (Fig. 2). Of the 557 *Al. philoxeroides* EDDMapS occurrence records, grand mean winter proportion mortality was 0.325 ± 0.120 (SD) for *Am. andersoni* and 0.770 ± 0.270 for *Ag. hygrophila*. Nominally, 91.8% of occurrence records in the USA fell within the combined highly and marginally suitable regions for *Am. andersoni*, compared to only 15.9% for *Ag. hygrophila*. The remainder of occurrences were in unsuitable regions, though no occurrences for *Am. andersoni* fell within regions predicted to be completely unsuitable, while 38.6% of alligatorweed occurrences were predicted to be completely unsuitable for *Ag. hygrophila.*

**Fig. 3.**
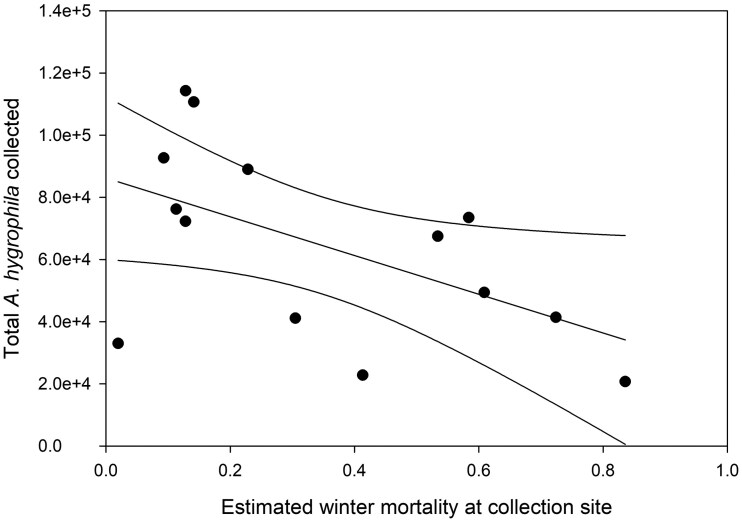
Regression of annual United States Army Corps of Engineers *Ag. hygrophila* collection data from 2001 to 2020 by estimated winter mortality at the collection site during the preceding winter. No collections were made from 2016 to 2018. Insufficient weather data were available for 2002, 2006, and 2019.

Predicted annual winter mortalities of *Ag. hygrophila* in Florida were correlated with USACE collection numbers the following spring (Fig. 3). For every percent increase in predicted mortality, the number of beetles collected in the spring was reduced by about 623.54 ± 279.09 (SE) (*t* = –2.23, *P* = 0.045, *r*^2^ = 0.294).

## Discussion

The history of *Alternanthera philoxeroides* biological control in the United States is an excellent case study to evaluate the consequences of climatic mismatches on biological control programs. Initial foreign exploration efforts were targeted to regions of South America, which were climatically similar to the USA Gulf Coast (Maddox et al. 1971). Putatively more cold-tolerant populations of *Ag. hygrophila* were later discovered in cooler regions of Argentina, released in 1979 to improve *Al. philoxeroides* biocontrol in temperate regions, and though they were successful at some sites, establishment was still restricted by winter temperatures (Buckingham and Boucias 1982). In the intervening decades, active redistribution efforts of *Ag. hygrophila* and unintentional spread of Al. philoxeroides and *Am. andersoni* have revealed the realized niche of each of these species within their introduced range. Nearly 30 yr after the predictions of Julien (1995), current model predictions describing the climatic mismatch between *Ag. hygrophila* and *Al. philoxeroides* are remarkably similar (Schmid et al. 2025).

There is significant value in the ability to make *a priori* predictions about establishment success and the potential range of introduced biological control agents. Especially when occurrence data are limited or nonexistent, mechanistic models provide a tool for making these predictions and could be particularly relevant for laboratory cultures, which differ substantially to natural populations. For example, because of inbreeding or other genetic bottlenecks experienced during importation and testing (Hopper et al. 1993). Current approaches that incorporate developmental data may undervalue the effects of thermal extremes, which are particularly limiting for tropical biological control agents. Use of the ULCIZ provides a tool for addressing lower thermal limits that gets around the limitations of other cold tolerance metrics (ie Overgaard et al. 2014).

The method described here, of applying ULCIZ to continuous temperature data to predict suitability, is a first attempt at incorporating these data into ecological niche modelling. We hope to introduce the metric for further consideration, but there is room to further develop the approach. It relies on several assumptions owing to either a lack of empirical data or sufficient acumen to incorporate these into more developed approaches to species distribution modelling. First, this current approach assumes chill injury is cumulative and that no recovery occurs between chill events, either on the individual or population level. On an individual level, there is evidence that insects can recover from chill injury during intermittent warming periods (Nedve˘D et al. 1998, Koštál et al. 2007). Recovery of insects at the population level will be influenced by developmental thresholds (Donohue et al. 2015), temperature-dependent development rates (Kuo et al. 2006), and the non-lethal effects of chilling on fitness (McDonald et al. 1997). As the model currently exists, it will over-estimate mortality unless recovery metrics or a threshold to bound calculations are incorporated.

Assessing climatic mismatches between tropical biological control agents and their hosts in temperate climates, which generally do not have specialized behaviors and physiologies for overwintering (Merkel et al. 2019), was the main goal of this study. This approach may still be useful for cold-adapted insects, however, special considerations for differences in cold tolerance by life stage would need to be made. In this study, adults of each species were used primarily because, as tropical insects with no obvious overwintering phenology, it was assumed that adults would be the primary overwintering life stage. This is somewhat supported by Stewart et al. (1999) who’s results indicate that the ULCIZ for different life stages of *Ag. hygrophila* may be associated with developmental thresholds, which range from 11.5 to 16.2 °C. The developmental thresholds and rates are not known for *Am. andersoni*.

Similarities between geographic ranges of *Am. andersoni* and *Ag. hygrophila* predicted by this approach and those predicted by Julien (1995) and Schmid et al. (2025) suggest that 1 mo may be an appropriate threshold, lacking additional incorporation of recovery or developmental information. Under ideal conditions, adults of both *Am. andersoni* and *Ag. hygrophila* can survive upwards of 2 mo; however, developmental times for each are approximately 28 d. A single month was chosen as the cut off for this study to encapsulate the estimated mortality on a single generation. This indicates that there very likely is some degree of recovery, on either the individual or population level, as mortality estimates would be substantially higher were this not the case. In the case of *Am. andersoni* habitat suitability in California, though both this study and Schmid et al. (2025) predict some suitability, particularly around Central California, our suitability predictions are quite a bit higher than Schmid et al. (2025), suggesting populations may limited by other climatic factors in this region. It would ultimately make the most sense to take the approach described here and incorporate it into existing mechanistic models, or vice versa.

Independent of ecological niche modelling, a temperature-by-time approach to assaying thermal limits is certainly a more holistic method for determining cold tolerance. Determination of the ULCIZ and SIT provides more comprehensive metrics for comparing cold tolerance among species than lethal temperature or exposure time experiments on their own. It should probably come as no surprise that the ULCIZ estimates for *Amynothrips andersoni* were lower relative to *Agasicles hygrophila*, as the former has been demonstrated to be more cold-tolerant with respect to chill coma and has a broader adventitious range within the United States (Knight and Harms 2022).

Though there is room for improvement and integration with existing ENM methods, there are several advantages of the current approach that have specific application to biological control programs. First, data on the native range of potential agents may be limited, constraining predictions made using correlative models. Further, the range of potential agents may be constrained by biotic or geographic factors unrelated to climate. Populations evaluated as biological control agents can also represent a very specific subset of the overall genetic diversity of a given species, and ranges predicted by correlative ENMs may not take into account intraspecific differences in thermal tolerances. The advantage of the proposed method, and mechanistic models in general, is that they provide a more targeted evaluation of available agent populations. Incorporation of the ULCIZ and SIT into mechanistic models provides an additional cold-tolerance metric, which may be particularly relevant for tropical biocontrol agents. Though the results of the proposed method are presented alone in this report, we ultimately hope others will build on these methods and incorporate them into existing models. It may also be improved through additional investigations into how individuals and populations recover from chill injury and respond to repeated exposure. We believe that with continued development the proposed method of predicting habitat suitability using experimentally derived cold-tolerance parameters will provide a valuable tool for improving new and existing biological control programs.

## Supplementary Material

nvaf099_Supplementary_Data
