# Supplementary material for: A proposed method for estimating habitat suitability of weed biological control agents with experimentally derived thermal injury and weather data
Source: Environ Entomol. 2025 Oct 1;54(6):1179–86. doi: 10.1093/ee/nvaf099 (PMC12716278; doi:10.1093/ee/nvaf099)
Supplement: nvaf099_Supplementary_Data [file nvaf099_supplementary_data.docx]

S1 Jacksonville District *A. hygrophila* collection data

| Year | Estimated winter mortality | Estimated total *A. hygrophila* collected | Lat | Long | Collection Dates |
| --- | --- | --- | --- | --- | --- |
| 2001 | 0.5341521 | 67500 | 29.0181 | -81.3106 | 5/7, 5/8 |
| 2003 | 0.6089851 | 49400 | 29.0181 | -81.3106 | 5/19, 5/20 |
| 2004 | 0.3046155 | 41100 | 29.425 | -81.5161 | 5/17, 5/18 |
| 2005 | 0.2282769 | 89000 | 29.0181 | -81.3106 | 5/9, 5/10, 6/9 |
| 2007 | 0.0928346 | 92700 | 29.0181 | -81.3106 | 4/30, 5/1 |
| 2008 | 0.5839669 | 73500 | 29.425 | -81.5161 | 5/6, 5/13, 5/19, 5/20, 6/11 |
| 2009 | 0.1413968 | 110700 | 28.6408 | -80.7308 | 5/14, 5/27, 5/28, 6/10, 6/17, 6/25 |
| 2010 | 0.7241455 | 41400 | 29.425 | -81.5161 | 6/9, 6/10 |
| 2011 | 0.8356897 | 20700 | 29.425 | -81.5161 | 6/7, 6/8, 6/9 |
| 2012 | 0.1278521 | 72300 | 29.425 | -81.5161 | 4/25 |
| 2013 | 0.4130413 | 22800 | 29.0181 | -81.3106 | 5/13 |
| 2014 | 0.1128925 | 76200 | 28.8147 | -81.2778 | 5/6, 5/7, 5/8, 5/13 |
| 2015 | 0.1281701 | 114300 | 28.8147 | -81.2778 | 4/6, 4/7 |
| 2020 | 0.0194013 | 33000 | 29.10734 | -81.4802 | 4/6, 4/7, 4/22 |
